# Emerging Role and Clinicopathological Significance of AEG-1 in Different Cancer Types: A Concise Review

**DOI:** 10.3390/cells10061497

**Published:** 2021-06-15

**Authors:** Sushmitha Sriramulu, Xiao-Feng Sun, Sarubala Malayaperumal, Harsha Ganesan, Hong Zhang, Murugesan Ramachandran, Antara Banerjee, Surajit Pathak

**Affiliations:** 1 Department of Medical Biotechnology, Faculty of Allied Health Sciences, Chettinad Academy of Research and Education (CARE), Chettinad Hospital and Research Institute (CHRI), Kelambakkam, Chennai 603103, India; sushi3biotech@gmail.com (S.S.); sarubala18@gmail.com (S.M.); harsha.scarlet@gmail.com (H.G.); rammurugesan50@gmail.com (M.R.); antara.banerjee27@gmail.com (A.B.); 2 Department of Oncology, Linköping University, SE-581 83 Linköping, Sweden; 3 Department of Biomedical and Clinical Sciences, Linköping University, SE-581 83 Linköping, Sweden; 4 Department of Medical Sciences, School of Medicine, Orebro University, SE-701 82 Orebro, Sweden; Hong.Zhang@oru.se

**Keywords:** AEG-1, biomarker, cancer, clinicopathology, inhibitor, pathway, therapeutics

## Abstract

Tumor breakthrough is driven by genetic or epigenetic variations which assist in initiation, migration, invasion and metastasis of tumors. Astrocyte elevated gene-1 (AEG-1) protein has risen recently as the crucial factor in malignancies and plays a potential role in diverse complex oncogenic signaling cascades. AEG-1 has multiple roles in tumor growth and development and is found to be involved in various signaling pathways of: (i) Ha-ras and PI3K/AKT; (ii) the NF-κB; (iii) the ERK or mitogen-activated protein kinase and Wnt or β-catenin and (iv) the Aurora-A kinase. Recent studies have confirmed that in all the hallmarks of cancers, AEG-1 plays a key functionality including progression, transformation, sustained angiogenesis, evading apoptosis, and invasion and metastasis. Clinical studies have supported that AEG-1 is actively intricated in tumor growth and progression which includes esophageal squamous cell, gastric, colorectal, hepatocellular, gallbladder, breast, prostate and non-small cell lung cancers, as well as renal cell carcinomas, melanoma, glioma, neuroblastoma and osteosarcoma. Existing studies have reported that AEG-1 expression has been induced by Ha-ras through intrication of PI3K/AKT signaling. Conversely, AEG-1 also activates PI3K/AKT pathway and modulates the defined subset of downstream target proteins via crosstalk between the PI3K/AKT/mTOR and Hedgehog signaling cascade which further plays a crucial role in metastasis. Thus, AEG-1 may be employed as a biomarker to discern the patients of those who are likely to get aid from AEG-1-targeted medication. AEG-1 may play as an effective target to repress tumor development, occlude metastasis, and magnify the effectiveness of treatments. In this review, we focus on the molecular mechanism of AEG-1 in the process of carcinogenesis and its involvement in regulation of crosstalk between the PI3K/AKT/mTOR and Hedgehog signaling. We also highlight the multifaceted functions, expression, clinicopathological significance and molecular inhibitors of AEG-1 in various cancer types.

## 1. Introduction

**Figure 1:**
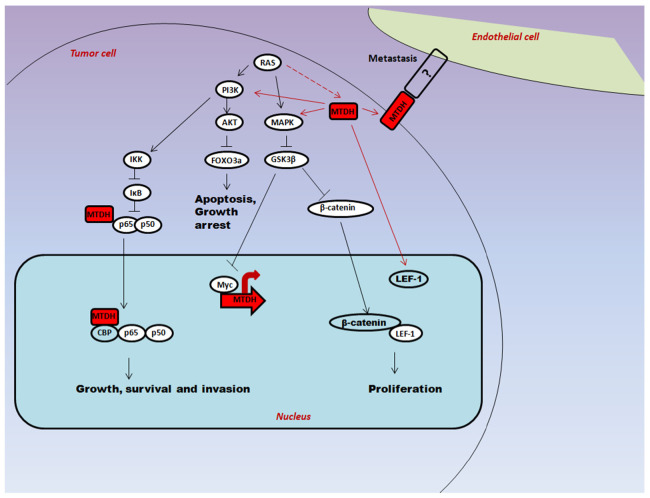
Involvement of AEG-1/MTDH in various signaling pathways (MTDH/AEG-1 enhances cancer progression through integration of several signaling pathways. The oncogenic *Ha-ras* increases *MTDH* expression through the activation of PI3K/AKT pathway, which phosphorylates and inactivates GSK-3β and subsequently elevates the stabilization and binding of c-Myc to the MTDH promoter. MTDH can activate AKT, NF-κB, and Wnt/β-catenin pathways to promote proliferation, survival, and invasion. Solid arrows indicate activation, bar-headed arrows indicate inhibition and dotted arrows indicate probable mechanism of action) [17].

Astrocyte elevated gene-1 (AEG-1)/Lysine-rich CEACAM1 co-isolated protein (LYRIC) in humans is encoded by gene metadherin *(MTDH)*; mapped in chromosome 8q22 is a prime oncogene, constituting about 528 amino acids, two nuclear localization sequences, an N terminal transmembrane region, a possible ATP or GTP-binding site and several putative phosphorylation sites [1], which is markedly overexpressed in several cancer types [2] as in esophageal squamous cell (ESCC) [3], gastric [4], colorectal [5], hepatocellular (HCC) [6,7], breast [8], prostate [9] and osteosarcoma [10]. AEG-1 helps in the progression and development of these cancers, and further, it was found that AEG-1 is necessitate in tissue proliferation, angiogenesis, invasion, metastasis and in reducing chemo- and radio-resistance. Oncogene *Ha-ras* works in the background for activation of AEG-1 through PI3K/AKT, which leads to AEG-1 transcriptional upregulation by c-Myc [11]. Once the *AEG-1* is overexpressed, it can trigger diverse oncogenic signaling cascades including PI3K/AKT, MAPK, Wnt, NF-κB and RNA interference (RNAi) (Figure 1) [12,13,14,15]. In previous studies, Brown et al. [16] utilized phage screening to exhibit MTDH-mediated metastases of the mouse breast cancer cells to lungs, thereby exemplifying the intrication of AEG-1 in cancer. Oncoprotein AEG-1 has been found to be involved in PI3K/AKT signaling, a pathway that controls the expression of several genes intricated in cancer cell survival, proliferation, invasion and metastasis [11]. On that account, we present a concise review on molecular mechanism of AEG-1 in the process of carcinogenesis and how it regulates downstream target proteins via crosstalk between the PI3K/AKT/mTOR and Hedgehog (Hh) signaling. In addition, we discussed the multifaceted functions, expression, clinicopathological significance and molecular inhibitors of AEG-1 in various cancer types.

## 2. Molecular Mechanism of Multifunctional Protein AEG-1 in Carcinogenesis

### 2.1. Oncogenic Ha-Ras Induces AEG-1 via PI3K/AKT Signaling

*Ras*, a proto-oncogene upon activation, initiates several complex signaling cascades, one of which is PI3K/AKT pathway, which has a pertinent role in cell survival [18]. Induction of *AEG-1* expression is achieved by Ha-ras and was further propitiated through PI3K/AKT signaling pathway. The mechanism of Ha-ras mediated tumorigenesis exemplified by Lee et al. [11] depicted a significant role of AEG-1 as a cancer promoter. It was observed from their research that Ha-ras was the causative for elevated expression of AEG-1, which is mediated by direct binding of c-Myc upon PI3K/AKT activation. The PI3K/AKT signaling is also found to be entwined in AEG-1-induced angiogenesis. In addition to the pro-angiogenic activity, it also empowers the neovasculature forming capacity of vascular endothelial cells [13]. PI3K/AKT was found to be necessary for *Ras*-induced *AEG-1* expression when shedding further light upon the molecular mechanism exemplifying *AEG-1* expression was attempted. Lee et al. [11] also expounded that AKT acts downstream to AEG-1 where activation of AKT led to GSK3B phosphorylation and repression of FOXO1/3 phosphorylation, which further ensued increased cell proliferation and survival.

### 2.2. Regulation of Downstream Target Proteins of PI3K/AKT Signaling by AEG-1

Several studies have shown that AEG-1 plays a noteworthy role in governing various pathological and physiological processes. Alteration in *AEG-1* expression levels due to certain cellular functions like invasion, metastasis, chemoresistance and angiogenesis has been reported in previous studies [14]. It is remarkable that most of the signaling pathways that have been identified thus far were revealed to be modulated by AEG-1 and they have also been linked with managing the inflammatory and immune responses [19]. AEG-1 has dealt a direct hand in enhancing the cell survival under diverse stimuli apart from tumorigenesis. Studies report that overexpression of *AEG-1* armors the cells against serum deprivation-induced injury and also play a pivotal role in PI3K/AKT pathway [20]. Under hypoxia, significant overexpression of *AEG-1* was bespoken and reactive oxygen species (ROS) stimuli in U87-MG cells heightened its ordination [21]. As reported by several researchers decode that AEG-1 can induce tumorigenic effects through PI3K/AKT pathway [3,22], Yin et al. [23] further investigated the molecular mechanism of lessened cell viability induced by decelerated *AEG-1* expression. Studies have illuminated upon the inhibition of classical PI3K/AKT pathway by silencing of *AEG-1*, and this decreased phosphorylated PDK-1 protein levels which were rightfully blamed for the aforementioned inhibition. Above study is parallel with the results reported by Kikuno et al. [24] where the knockdown of *AEG-1* downregulates phosphorylated PDK-1, though the underlying mechanism is not clear. Phosphorylated PDK-1, in turn phosphorylates and activates AKT. Hu et al. [25] demonstrated a direct interaction between AEG-1 internal domain and AKT2 PH domain, and this interaction caused extension of AKT2 phosphorylation stability by controlling the downstream signaling pathways. Hence, feedback between AEG-1 and PI3K/AKT pathway concur to reduction in cell viability, making AEG-1 a central player. Furthermore, the findings by Yin et al. [23] suggested that phosphorylated AKT was required for CREB activation as overexpression of *AEG-1* was linked with elevated phosphorylated CREB expression through PI3K/AKT pathway.

### 2.3. Crosstalk between the PI3K/AKT/mTOR and Hedgehog Signaling

From previous studies, it is well-known that PI3K/AKT signaling pathway is a commonly activated pathway that has crosstalk with several other oncogenic pathways. PI3K/AKT/mTOR pathway with a significant role in proliferation, angiogenesis, survival, and differentiation has been shown to modulate GLI activity in normal and tumor cells [26]. Crosstalk between Hh and PI3K/AKT/mTOR and upregulation of Hh proteins was observed in esophageal models [27,28]. In esophageal tumor models, correlation of AKT activation and upregulation of Hh target gene has been decoded. This PI3K/AKT/mTOR pathway plays a key role in SHH-induced Hh signaling where this PI3K/AKT pathway concord with Hh at the GLI1 level to further encourage cellular proliferation and survival of esophageal cells [29]. A phosphoglycoprotein named Osteopontin, expressed strongly in tumor stromal cells, activated *GLI1* expression and transcriptional activity by domineering AKT-GSK3β. Several studies have proved that PI3K/AKT/mTOR pathway and Hh pathway interact with each other for tumor metastasis enhancement by induction of epithelial-mesenchymal transition (EMT) [30]. In a mouse xenograft model of human gastric carcinoma, expression of SHH increased the metastasis through PI3K/AKT pathway activation. This pathway inhibition prevented SHH-induced EMT and further halted cancer invasiveness and metastasis [31].

**Figure 2:**
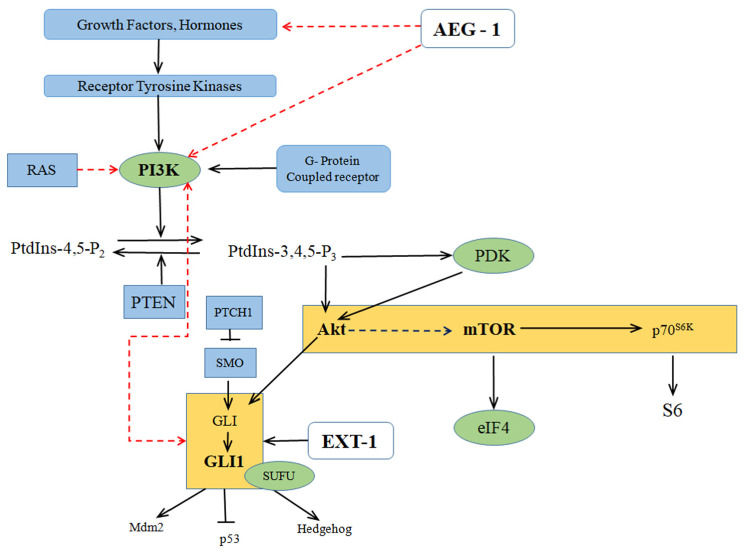
Involvement of AEG-1/MTDH and EXT-1 in PI3K/AKT/mTOR and Hh signaling pathways (Oncogenic *Ha-ras* activates MTDH through the PI3K/AKT pathway. Crosstalk between PI3K/AKT/mTOR and Hh leads to tumorigenesis. Solid arrows indicate activation, bar-headed arrows indicate inhibition and dotted arrows indicate probable mechanism of action) [42].

GLI/Hh signaling pathway has a dignified role in cancers’ initiation and progression where it administers several oncogenic events including proliferation, survival and metastasis [32,33]. Activation and regulation of complex Hh signaling occurs at multiple levels within a signaling cascade. SHH ligand-PTCH receptor binding inaugurates canonical Hh signaling. PTCH interaction with Hh diminished the hindrance effect on smoothened (SMO) transmembrane protein. De-oppressed SMO triggers the GLI transcription factors which further get activated, those GLIs turn on genes’ transcriptional activity imprinted in cell survival, cell cycle and stemness [34,35]. Non-canonical regulation of Hh signaling involves interference with oncogenic PI3K/AKT/mTOR pathway, which also has modulation capacity of Hh signaling output. Several studies have reported that protein translation components were lorded over by Hh signaling via PI3K/AKT/mTOR pathway [36]. Crosstalk between mTOR and Hh was present in several systems and it has been hypothesized that mTOR and Hh are entangled in the metastasis process of various cancers [37]. PI3K/AKT/mTOR pathway positively regulates Hh signaling via elevating the activity of GLI1, GLI2 and GLI3. Recent studies report the molecular mechanism for the interference between PI3K/AKT/mTOR and SHH. Hh activating mutations of PTCH and SUFU resulted in increased PI3K signaling [38]. Studies from our lab also reported that silencing *AEG-1* impedes proliferation and decreases invasive potential in colon cancer cell lines through interaction with Exostosin-1 *(EXT-1)*, a well-known tumor suppressor of Hh signaling pathway, which plays a monumental role in chain elongation step of heparan sulfate biosynthesis, simultaneously upregulating it whilst AEG-1 was silenced [39]. We have also conferred from our recent studies that PTCH-1, which is found to be involved in Hh pathway is a direct interactor of AEG-1 [40], and this PTCH-1 has been found to interact with EXT-1 [39], thus confirming that *AEG-1* silencing might modulate *EXT-1* expression through the crosstalk between PI3K/AKT/mTOR and Hh signaling cascades. Previous reports suggest that Hh signaling regulates AKT target genes expression, where AKT1 was the direct transcriptional target of GLI1. Escalated PI3K/AKT/mTOR signaling leads to increased Hh signaling by heightening the activity of Hh transcription factors GLI1, GLI2 and GLI3 [41]. Involvement of AEG-1 in crosstalk between the PI3K/AKT/mTOR and Hh signaling has been illustrated in Figure 2.

From the emerging studies, it has also been proven that combined targeting of PI3K/AKT/mTOR and Hh pathways provide great therapeutic value in medulloblastoma treatment. Several molecular and pre-clinical studies have impressed upon the atypical activation and crosstalk between Hh and PI3K/AKT/mTOR signaling cascades leads to chemoresistance and tumorigenesis [43]. Chaturvedi et al. [44] proved that combined targeting of these two pathways using small molecule inhibitors exhibited anti-tumor effects in medulloblastoma in vitro and in vivo. These observations solidify the crosstalk between Hh signaling and PI3K/AKT/mTOR. Subsequent signaling effects and AEG-1 interaction with other proteins need to be construed. Even though how various signaling cascades commingle in mediation of oncogenic functions of AEG-1 has been understood, not much is known on the direct interactions of AEG-1 with other downstream target proteins [19]. Nevertheless, demystifying the potential biological functions of AEG-1 and its mechanism of action in different cancer types remains to be achieved.

## 3. Multifaceted Functions of AEG-1 in Cancer Progression

Studies have more clearly exemplified that AEG-1 plays a predominant role in the proliferation, angiogenesis, invasion and metastasis of cancers by either overexpressing or repressing the specific gene [45].

### 3.1. Proliferation

Proliferation of malignant tumors is an outcome of acquisition of limitless growth capacity and resistance to cell death. In some studies, it has been demonstrated that AEG-1 enhances serum independent cell growth by impeding serum starvation-induced cell death [20]. It is explained that in non-small-cell lung carcinoma (NSCLC), AEG-1 could impede apoptosis via elevating the anti-apoptotic protein BCL-2 level and also through activating PI3K/AKT pathway [46]. According to the studies mentioned above, there are more evidences to prove that AEG-1 has anti-apoptotic potential. AEG-1 in combination with Ha-ras can increase the colony forming ability of human astrocytes and immortalized normal human melanocytes, propounding a possible role in tumorigenesis [47]. Overexpression of *AEG-1* can significantly increase proliferation and anchorage-independent growth potential in several types of cancers such as ESCC [3], HCC [6], gallbladder cancer (GBC) [48], breast [22], glioma [49] and so on. Studies of Liu et al. [50] showed that suppression of AEG-1 ensued cell cycle arrest in the G0/G1 phase of cell cycle and further impeded colony forming ability and stimulated apoptosis in neuroblastoma cells. Immunohistochemical analysis is used to scrutinize AEG-1 expression in normal tissue, atypical ductal hyperplasia, usual ductal hyperplasia, ductal carcinoma in situ (DCIS) and invasive carcinoma in the breast. Results exhibited that AEG-1 was not much expressed in normal cases, but with advancement of intraductal proliferative lesions, intensity of expression seems to be higher. Increased expression of DCIS cases may propound that AEG-1 plays a significant role in initiation of ductal carcinoma. It was also observed that overexpression of *AEG-1* is significantly correlated with increased Ki67, which is a potent proliferative marker [51]. Further, inhibition of AEG-1 by retaining microRNA-30a (miR-30a) can reduce proliferation, clonogenic ability, and invasion of breast cancer [52].

### 3.2. Angiogenesis

Angiogenesis is an elemental event in the perpetuation and advancement of tumors and their metastases. Interaction of pro-angiogenic factors, matrix metalloproteinase (MMP) and angiogenic inhibitors controls the process of angiogenesis [53]. In Immunohistochemical analysis, tumors procured from nude mice were injected with cloned rat-embryo fibroblasts AEG-1 clones and demonstrated that tumors have increased microvessel density and levels of markers of angiogenesis, including ANG-1, MMP-2, and hypoxia inducible factor 1-α. In chicken chorioallantoic membrane (CAM) angiogenesis model, suppression of *AEG-1* made glioma cells display neo-vascularization. In vitro angiogenesis assays also indicated that AEG-1 elevates the tube formation in human umbilical vein endothelial cells, which is a significant framework of endothelial function in angiogenesis [13]. Studies discussed above confirm that AEG-1 has a dominant function in modulating angiogenesis. AEG-1 is also found to promote angiogenesis in human HCC [7]. In addition, analysis of specimens from 125 patients with breast cancer exhibited that AEG-1 correlated positively with VEGF levels [54].

### 3.3. Metastasis

Evolving cancer possess metastatic potential which is a multifactorial and multistep process involving a vigorous interplay between several metastasis-associated genes which evoke changes in various signal transduction pathways, and encode different adhesion molecules, extracellular matrix protein hydrolases, angiogenic factors, etc. Studies demonstrate that AEG-1 is notably relevant with invasion and metastasis of cancers. In Matrigel invasion assays, tumor cells, for example, Hela cells, HCC, glioma cells and neuroblastoma cells [55], exhibited markedly increased invasive ability by overexpression of *AEG-1*. Previous studies confirm that AEG-1 is successively pertinent in metastasis of breast cancer. Overexpression of *AEG-1* not only elevates lung metastasis but also led to a modest elevation of bone and brain metastasis and shortened the mice survival when injected with breast cancer cells [56,57]. Conversely, *AEG-1* suppression significantly decreased the adherence of breast cancer cells to lung micro vascular endothelial cells and to bone marrow to some extent [58]. According to studies by Zhou et al. [59] AEG-1 enhanced lung metastasis of HCC by increasing orientation chemotaxis and adhesion abilities of HCC cells. Furthermore, AEG-1 plays a pivotal role in cancer metastasis, which is also discerned in CRC, renal cell carcinoma, GBC, prostate and ovarian cancer [60,61,62,63,64]. Recently, it has been found that EMT is linked with the invasion and distant metastasis of tumors. Transition of epithelial cells into mesenchymal cells lose polarity, increase the metastatic potential and upregulate nuclear expression of various transcription factors along with loss of epithelial phenotype markers, such as the marker E-cadherin and acquisition of mesenchymal markers vimentin and β-catenin, which are cytoskeletal proteins [65,66]. Several reports have exhibited that controlling the *AEG-1* expression can mediate EMT of a tumor cell. Overexpression of *AEG-1* increased the invasion of breast cancer cells, along with conversion from tightly packed cobblestone-like appearance to loosely packed spindle like morphology, which is an exact characteristic of mesenchymal cells. Upregulation of *AEG-1* manifested that epithelial marker E-cadherin was decreased, while the mesenchymal markers including vimentin/fibronectin, and certain other transcription factors like snail and slug were increased [67]. Ward et al. [68] established a tamoxifen-resistant (TamR) breast cancer model, which exhibited mesenchymal properties with decreased *E-cadherin* expression and increased expression of *fibronectin* and *ZEB1*. Studies suggest that AEG-1 enhances the EMT of breast cancer, and also plays an important role in chemoresistance. Certain studies revealed that AEG-1 is involved in ursolic acid-mediated EMT inhibition of lung cancer [69]. Moreover, it is confirmed in HCC, osteosarcoma and head-neck squamous cell carcinoma (HNSCC) that AEG-1 is able to increase metastasis of tumor by instigating EMT of tumor cells [70,71,72]. Also, AEG-1 overexpressed *tetraspanin* and *Claudin 4*, which thereby increases invasion [6]. Thus, it can be presumed that overexpression of *AEG-1* may negatively impact apoptosis and cell cycle-checkpoints and thereby increases cell survival [73].

## 4. Expression of AEG-1 in Different Cancer Types

### 4.1. Esophageal Carcinoma

Esophageal carcinoma is of two distinct types: ESCC and esophageal adenocarcinoma (EAC) with variant etiological and pathological characteristics transpiring at distinct geographical areas throughout the world [74]. There is no noteworthy improvement in prognosis, with a 5-year survival rate of about 10–20%. It was shown in a study that markedly overexpressed *AEG-1* could significantly raise the proliferation and anchorage-independent growth potential. Whereas the *AEG-1* suppression by RNAi leads to hindrance in cell proliferation and anchorage-independent growth potential on soft agar assays. In another study, it was demonstrated that increased expression of *AEG-1* could eventually leads to suppression of *p27^Kip1^* and *cyclin D1* through AKT or FOXO3a pathway [75]. AEG-1 urges human fetal astrocytes which is infected by HIV-1/treated with gp120/tumor necrosis factor and has evidence related to tumor initiation and induction of metastasis [76]. The prognosis of ESCC depends upon conventional pathological variables like tumor size, tumor grade, lymph node and distant metastasis status. Hence, there is a significant need of sensitive and specific biomarker for better therapeutic strategies in cancer patients.

### 4.2. Gastric Cancer

Gastric cancer is the third leading cause of death globally and its prevalence varies in distinct geographical areas. AEG-1 can localize at various cellular parts as in tight junctions, nucleus, endoplasmic reticulum, nucleolus, perinuclear region, cytoplasm and cell surface, but the relation between the localization and its function is not clear [77]. Studies have exemplified that inhibition of expression of *AEG-1* by specific siRNA, inhibited SCG-7901 growth and elevated apoptosis. Reduced AKT phosphorylation, GSK3B (ser9) and cyclin D1 decrease the β-catenin level and lymphoid enhancer binding factor 1 (LEF1) due to inhibition of AEG-1. The *AEG-1* overexpression elevates angiogenic factor production, including angiopoietin-1, hypoxia-inducible factor 1 alpha (HIF-1α), Tie2 and MMP-2. Resistance of cancer to several chemotherapeutic agents can be seen due to the role played by AEG-1 in cancer [78]. Despite of highly advanced operative measures and novel drugs, still gastric cancer continues to be the massive problem. Hence, identification of sensitive and novel biomarker for prognosis is in high demand [6,13,79,80].

### 4.3. Colorectal Cancer

Localization of AEG-1 at nuclear rim, perinuclear region, cytoplasm and the endoplasmic reticulum was demonstrated by immunohistochemistry and immunofluorescence studies [81]. In a study, it was shown that AEG-1 pathway activation leads to overexpression of *Ha-ras* in human astrocytes. Immortalized using Simian Virus 40 T/t antigen and Human telomerase reverse transcriptase (hTERT) led to overexpression of *AEG-1* [11]. Moreover, the study showed that it is stimulated by PI3K/AKT pathway, and further the transcriptional factor c-Myc binding to AEG-1 promoter region. An anti-apoptotic and pro-proliferative gene are triggered on by silencing NF-κB inhibitor, which in turn raised binding of NF-κB-DNA binding activity and allowed the migration of NF-κB from cytoplasm to nucleus which thus physically gets interacted with NF-κB subunit p65 and modified the function. Hence, *AEG-1* is overexpressed at mRNA and protein level [2] and involved in CRC progression and aggressiveness through NF-κB signaling pathway [82]. This AEG-1 plays an important role in activation of various signaling pathways involved in CRC proliferation and invasion [39].

### 4.4. Hepatocellular Carcinoma

AEG-1 is the key contributor in this HCC malignancy. The major risk factors were identified as high alcohol consumption, hepatitis B and C viruses (HBV and HCV) infection, and aflatoxin B1 exposure. In recent studies on HCC, it was observed that there was overexpression of *AEG-1* in tumor cells when compared to the normal cells. Current standard surgical procedures, drugs and liver transplantations are under satisfactory [83]. Thus, there is need of biomarker for prognosis so that the sufferings of the patients can be reduced and helps in the treatment.

### 4.5. Gall Bladder Carcinoma

Despite of implementation of highly improved surgical techniques and chemotherapeutic drugs, the clinical outcome of GBC patients remains inadequate as around 90% of patients die due to advanced diagnosis stage where the surgical procedures and drugs could not save the lives efficiently. Presently, surgical removal of the gall bladder along with removal of some parts of liver and lymph nodes is the regular treatment. In a study, it was demonstrated that overexpression of *AEG-1* in two cancer cell lines of gall bladder (GBC-SD and SGC-996) are responsible for initiation and progression of cancer and leads to aggressive state metastasis, which becomes the initial stage to cause secondary cancers. Thus, there is need to overcome the poor prognosis by discovering biomarkers [84,85,86].

### 4.6. Breast Cancer

Recently, the incidence of breast cancer has elevated dramatically with respect to cancer-associated morbidity [87,88,89]. Statistics reveal that the breast cancer with *AEG-1* overexpression positively correlated with clinical conditions and metastasis. Wnt signaling is closely related to diversity of the tumor development and plays a key role in metastasis of breast cancer [90,91,92,93]. Wnt signaling activation mechanism in various cancer types are yet to be studied and identified. β-catenin is the central player in regulation of Wnt signaling and found to be actively associated with the disease progression and poor prognosis in breast cancer [94]. During the activation of the Wnt signaling pathway, β-catenin assembled in the cytosol at high rate, which further leads to the activation and expression of target genes such as *cyclin D1*, *MMP-9* and *c-Myc* [95,96,97]. Wnt signaling pathway is said to be active when the β-catenin accumulation is more in the nucleus.

### 4.7. Prostate Cancer

In prostate cancer, the HIF-1α plays an important role in proliferation, angiogenesis, erythropoiesis and androgen resistance. Radical prostatectomy and androgen ablation therapy are the most common treatments in initial stages. In a study, an interesting fact was found that when *AEG-1* was knockdown, it induces cellular apoptosis by increasing FOXO 3a activity. Increased expression of *AKT* associates with metastasis in different cancer types including prostate cancer. Induction of the inhibition of apoptosis by various mechanisms involves conformational changes in *Bad*, *Bax* and *Caspase-9* directly by activation of AKT. Indirect apoptosis is acquired by impacting the activities of various transcription factors like FOXO 3a and NF-κB. Studies demonstrated that overexpression of *AEG-1* induces angiogenesis and metastasis, further leading to aggressive carcinoma. Thus, AEG-1 may be a significant biomarker for predicting and improving prognosis of prostate cancer patients [24,98].

### 4.8. Non-Small Cell Lung Cancer

Over 20 years, effective surgical techniques have been observed, including radiotherapy and new chemotherapeutic agents, but the consequence of lung cancer has not been noticeably improved. Non-small cell lung cancer (NSCLC) patients die due to failure of the therapy, which in turn enhances metastasis. In most cases, the disease is diagnosed in advanced stages mostly when the cancer has extended to other organs of the body. Initially, there is a loss of intracellular adhesion, degradation of the extra cellular matrix and migration, which leads to tumor cell mobility and invasive ability. PI3K/AKT and NF-κB signaling transduction pathways were found to be activated by overexpression of *AEG-1*, and were linked with the early progression of metastasis [99]. Clinically, the prognostic importance of AEG-1 expression was only attracting attention in recent years, which was reported in NSCLC [11,20,100].

### 4.9. Glioma Cancer

Malignant glioma demonstrates the fused tissue invasion, with aggressive tumor growth and neurodegeneration being the significant hallmarks. Increased expression of AEG-1 is seen in various cancer types and over more than 90% are brain tumors. In recent studies, it was noticed that when AEG-1 synergize with Ha-ras, promotes the clonogenic ability of immortalized non-tumorigenic melanocytes and leads to augment in invasion of tissue and thus act as positive feedback activator molecule. The current treatment involves debulking surgery, along with chemotherapy cycles and radiation therapy. Even with these therapeutic regimens and procedures, the prognosis remains poor. There is an alert requirement of novel therapeutic strategies to arrest the signaling cascades responsible for tumor formation and to improve the treatment process [101,102].

### 4.10. Osteosarcoma

Osteosarcoma is the eighth common type of carcinoma and is the most frequent cause of death worldwide. AEG-1 and endothelin-1(ET-1)/endothelin-A receptor (ETAR) have significant signaling cascade role in osteosarcoma and also plays a pivotal role in invasion and chemoresistance [103]. *AEG-1* overexpression in Saos-2 cells leads to increased expression of ET-1 both at transcriptional and translational level, MMP-2 expression and cell survival in case of cisplatin. Removal of these effects was achieved by using PI3K inhibitor, ETAR inhibitor, Ly294002, and BQ123. Decreased expression of ET-1, cell invasion MMP-2 expression and cell survival against cisplatin were seen when *AEG-1* was suppressed in MG-63 cells [104,105]. It was shown in a study that *ET-1* expression at the transcriptional level in osteosarcoma cells was regulated by AEG-1 in PI3K-dependent manner.

## 5. Overview on Clinicopathological Significance of AEG-1 in Different Cancer Types

Clinical analyses demonstrated that there is a significant correlation between AEG-1 expression and certain clinicopathological parameters, which include lymph node metastasis, differentiation, stage, recurrence and survival of patients, and therapeutic responses. In FFPE sections of 168 ESCC patients, the IHC analysis revealed 92.9% were positive cases of AEG-1 and it was overexpressed in tumor tissues compared to adjacent normal tissues [3]. As there is an inverse correlation between AEG-1 and overall survival, AEG-1 suggested to serve as a potential prognostic marker in ESCC [3]. In gastric cancer, IHC analysis revealed cytoplasmic AEG-1 overexpression in 66 clinical cases out of 105 gastric cancer patients. Patients with AEG-1 overexpression had 23 months of median overall 5-year survival rate and 38 months for AEG-1 negative patients [4]. Significant overexpression of AEG-1 was also observed in tumor tissues compared to adjacent normal tissues in 119 gastric cancer patients [106] suggesting the potential of AEG-1 as a prognostic marker (*p* < 0.05) in gastric cancer. Globally, CRC accounts for about 1 in 10 cases of cancer and cancer-related deaths [107]. Analysis of adjacent normal tissues, tumor and metastatic liver samples in 520 CRC cases revealed overexpression of AEG-1 in liver metastasis [60]. From another study, analysis by qRT-PCR in 156 CRC cases showed mean AEG-1 mRNA level was 371.56 ± 348.37 in primary cancer tissues and 214.98 ± 156.39 in adjacent normal tissues. Similarly, AEG-1 protein expression was analyzed in this study by IHC which showed overexpression of AEG-1 protein in primary CRC tissues compared to normal tissues [2]. AEG-1 might serve as a prognostic marker in CRC as all studies exhibited that level of AEG-1 increases with CRC progression and negatively correlate with overall survival [108]. In HCC, IHC analysis of 323 patients exhibited significant AEG-1 overexpression in 54.2% of the patients. In addition, the overall survival in high AEG-1 expression group is lower compared to the low AEG-1 expression group (HR = 1.870, *p* < 0.001) [70]. Similarly, in another study, IHC analysis of 85 HCC samples concluded that AEG-1 (HR, 4.756, 95% CI, *p* = 0.003) might serve as a prognostic factor [109]. Recent studies on 71 GBC samples demonstrated that overexpression of AEG-1 correlated with reduced overall survival which also further revealed that AEG-1 expression (*p* = 0.011) is an independent prognostic factor of GBC [110]. Through DNA microarray analysis of 117 breast cancer patients, a cluster of 4968 significant genes were identified and found to be linked with prognosis, out of which AEG-1 is the 25th most correlated gene [111]. In IHC analysis of 225 breast cancer patients, 93.3% of patients were AEG-1 positive among which 44.4% were classified as high expression group [112]. In another study, IHC analysis of 170 breast cancer patients demonstrated high AEG-1 expression in 47% cases which is found to be associated with shorter survival (*p* = 0.0008) [58]. In a tissue microarray containing 63 benign prostatic hyperplasia, 11 prostate cancer bone metastasis and 143 prostate cancers, AEG-1 expression was analysed by IHC which revealed that the expression was found to be higher in prostate cancer compared to benign prostatic hyperplasia (*p* = 0.037). Nine out of eleven bone metastasis patients showed AEG-1 overexpression in cytoplasm as well as in cell membrane. Nuclear AEG-1 was detected in 38 out of 143 prostate cancer cases in which the mean survival was 70 months in nuclear AEG-1 positive and 39 months in nuclear AEG-1 negative patients [113]. This study confirms that in addition to expression, AEG-1 localization might serve as a prognostic marker in prostate cancer. Reports have displayed that the IHC analysis of 95 NSCLC with non-lymphatic metastasis, 105 with lymphatic metastasis and 20 distant metastasis patients showed AEG-1 overexpression in all cancer tissues compared to adjacent normal tissues which showed less expression (*p* < 0.001). AEG-1 overexpression was inversely correlated with overall survival (*p* < 0.001) which thereby confirms that AEG-1 serves as an independent prognostic factor for patient outcome in NSCLC patients [114]. IHC analysis which was performed in 296 glioma patients also demonstrated that 89.5% were AEG-1 positive cases among which 51.7% were identified with high AEG-1 expression and 48.3% with low expression (*p* < 0.001). Increased AEG-1 expression was found in patients with greater than 45 years of age (*p* < 0.001) [115]. Analysis of 204 tissues demonstrated AEG-1 positivity in 74.4% of tumor cases [116]. In another study, IHC analysis on 86 human glioma cases revealed that there is a progressive increase in the levels of both AEG-1 and MDM2. A positive correlation between AEG-1 and MDM2 was observed and their expression levels were correlated with poor overall survival concluding AEG-1 and MDM2 as significant prognostic factors for glioma [117]. Analysis of IHC in 62 osteosarcoma patients and 20 normal bone samples revealed AEG-1 positivity in 82.3% of cases out of which 32 cases exhibited increased AEG-1 expression. Overall survival was 57.188 months (95% CI, 44.608–70.308) in high AEG-1 expression group and 91.73 months (95% CI, 77.950–105.511) in less AEG-1 expression group. Interestingly, the levels of AEG-1 were higher in the female patients (*p* = 0.018) [57]. These studies identified AEG-1 as an independent prognostic factor in osteosarcoma patients. It has been well established that AEG-1 might be an independent prognostic biomarker in various types of cancers including the tongue [118], bladder [119,120], breast [112,121], endometrial [122] and ovarian cancers [123]. High AEG-1 expression was also recorded in tumors of salivary gland and is further linked with poor overall survival. Furthermore, MammaPrint which contains a unique 70-gene signature, including AEG-1 is the first and only Food and Drug Administration (FDA) approved personalized metastasis risk analysis assay for breast cancer. The serum samples from 483 breast cancer patients and 230 normal donors were detected by ELISA for search of new biomarkers in early diagnosis. Results indicated that anti-AEG-1 antibodies have a substantial correlation with the age and stage of patients thereby, AEG-1 may be utilized as a novel biomarker for the prognostic evaluation of cancer patients with AEG-1 positive expression [124].

## 6. MicroRNAs and Silencing RNA as Effective AEG-1 Inhibitors

Molecular inhibitors participate in the restriction of downstream functions following the interaction with the target of interest, in case of AEG-1, a range of miRNAs have been reported to inhibit the protein expression (Table 1). One such miRNA is miR-195, a well documented miRNA playing a role in cancer associated pathways is also involved in inhibition of AEG-1 activity eventually restricting cell proliferation in HCC [125]. HCC progression could be further restricted by AEG-1 inhibition, not just by miR-195, but also miR-875-5p [126]. Similarly, in HCC exhibiting higher sorafenib resistance, the condition could be partially reversed by the degradation of AEG-1 through interaction with miR-375 in the cancerous cells [127], hence paving way for better sorafenib treatment in HCC patients. Studies have also proved that miR-342 is one of the targets of AEG-1. Overexpression of *AEG-1* downregulated this particular miRNA concluding that it is a tumor suppressor miRNA [128]. MiRNAs like miR-375, miR-195, miR-497 and miR-136 was shown to negatively regulate the AEG-1 in HCC [129]. Overexpression of miR-497 inhibited the metastasis in HCC by targeting AEG-1 [129]. Similarly, downregulating miR-136 led to increased expression of *AEG-1* which in turn increased cell survival and proliferation in HCC [130]. Overexpression of miR-377 and miR-874 markedly inhibited the cell proliferation and migration by interacting with 3′ untranslated region of AEG-1 in NSCLC and retinoblastoma respectively [74,131]. Yang et al. [132] demonstrated the interaction of AEG-1 with miR-136 and thus targeting miR-136 decreased the chemo-resistance and induced apoptosis in glioma. Reports in cervical cancer samples proved that miR-124 targets AEG-1 and inhibits the EMT [133]. Forced expression of miRNA-217 in colon cancer cell lines led to significant reduction of AEG-1 mRNA and protein expression. Similarly, inhibition of miR-217 in normal colon epithelial cells caused elevation in AEG-1 levels, implicating its importance as a prognostic marker and confirming the mRNA-miRNA interaction [134]. Being associated with a wide range of cancers, AEG-1 has a pivotal role on inhibition of cancer progression in various cases such as breast cancer when it interacts with miR-30 [52], retinoblastoma, where the interaction is between AEG-1 and miR-504 [135]. Following the analysis of laryngeal cancer, miR-448 exhibited restriction of cell migration, proliferation and other metastatic conditions [136] and in the case of malignant glioblastoma, the compound DYT-40 has reported to activate the mitochondrial apoptosis pathway hence inhibiting AEG-1 [137]. Other than miRNAs, a wide variety of natural compounds such as Cryptotanshinone and PB0412-3 (PB3) restricted metastasis in hypoxic prostate cancer and brain tumour respectively [9,138]. Currently, RNA silencing (siRNA) has proven to be effective in inhibition of AEG-1 as exhibited by the vector based short hairpin RNA (shRNA) in pancreatic cancer [139] and RNAi as revealed in cervical cancer [140].

Discovering new AEG-1 inhibitors for cancer treatment through in vitro assays using 3D models is necessary as majority of the studies are based on transcriptomic profiling of cells which are grown in 2D. New lines of research have discussed the variability of genetic and protein profiling in 2D vs. 3D. Future studies can benefit from 3D self-organized models and organ-on-a-chip platforms for advanced imaging where spatial transcriptomics can be applied in combination with other imaging modalities such as expansion microscopy [141,142]. In case of above mentioned limitations, future directions such as the humanized model and the use of advanced spatial transcriptomic techniques such as MERFISH enable the recognition of rare populations of cells and the characterization of transcriptionally distinct cell types within huge tissue regions [143,144].

In view of the fact that overexpression of AEG-1 is found in different types of cancers and it controls the process of carcinogenesis, inhibiting AEG-1 might be an effective anti-cancer strategy. Formulating potent therapeutic strategies based on the drugs that are currently in use or in evolution which might impact the AEG-1 function is necessary in order to generate AEG-1 inhibitors [14]. Enormous efforts are made in developing PI3K/AKT inhibitors and there are more than nine PI3K inhibitors that are being assessed clinically and exhibiting anti-cancer efficacy [145]. A proteasome inhibitor named Bortezomib, led to the successful entry of its utility in cancer indications [146]. Combination of PI3K inhibitors and Bortezomib might act as a therapeutic strategy to impede cancer phenotypes that are induced by AEG-1 [145]. Thus, developing a clear understanding on the underlying molecular pathophysiology of cancers will impart several opportunities to generate novel treatments in near future. This can be achieved by incorporation of high-throughput screening techniques [147] and computational tools like CADD [148] which can have a significant impact on drug discovery by reducing both time and cost and minimizing follow-up risk with the development of non-viable leads.

## 7. Conclusions

The predominant role of AEG-1 in controlling signaling pathways of several cancers displays that it has a major role in cancer progression and metastasis. Oncogenic *Ha-ras* activates AEG-1 expression and further this AEG-1 regulates the defined subset of its downstream target proteins of various oncogenic signaling pathways. Triggering AEG-1 expression activates several other downstream signaling pathways including PI3K/AKT/mTOR and Hh cascades. As the effect on AEG-1 modified the proteins in other signaling pathways directly, this confirms the existence of crosstalk between PI3K/AKT/mTOR and Hh. Silencing *AEG-1* expression might inhibit the downstream target proteins of PI3K/AKT/mTOR and Hh which in turn might prevent metastasis. Functional and clinical analyses have established that AEG-1 could be considered as a potent target in the treatments of cancers. Nevertheless, mechanisms of this multifunctional gene in modulating carcinogenesis remain poorly understood, and the findings are still necessary to validate. Contribution of AEG-1 to chemo- and radiotherapy resistance provides an advanced direction for evolution of anti-cancer therapies. Meanwhile, deeper understanding of canonical functional domains of AEG-1 is expected to enable the development of small molecule target inhibitors. Though there are evidences that AEG-1 is increased in many cancer types, it is significant to find out whether AEG-1 represents a viable target for cancer therapies. Future investigations might promote AEG-1 as a pivotal tool that could be ordinarily employed in diagnosing cancers, observing the progression of cancer and assessing the efficacy of treatment.

## Figures and Tables

**Table 1:** List of miRNAs and siRNA as AEG-1 inhibitors in different cancer types.

AEG-1 Inhibitors	Type of Molecule	Associated Conditions	Reference
miR-195	miRNA	Hepatocellular carcinoma	[125]
miR-875-5p	miRNA	Hepatocellular carcinoma	[126]
miR-375	miRNA	Hepatocellular carcinoma	[127,129]
miR-136	miRNA	Hepatocellular carcinoma, Glioma	[130,132]
miR-874	miRNA	Retinoblastoma	[131]
miR-124	miRNA	Cervical cancer	[133]
miR-217	miRNA	Colorectal cancer	[134]
miR-30	miRNA	Breast cancer	[52]
miR-504	miRNA	Retinoblastoma	[135]
miR-448	miRNA	Laryngeal cancer	[136]
DYT-40	Novel synthetic compound	Malignant glioblastoma	[137]
PB0412-3 (PB3)	small molecule polyheterocyclic compound	Brain tumor	[138]
Cryptotanshinone	Natural compound (abietane-diterpene derivative)	Prostate cancer- Hypoxia	[9]
shRNA	shRNA	Pancreatic cancer	[139]
RNAi	siRNA	Cervical cancer	[140]
miR-377	miRNA	Non-small cell lung cancer	[74]

## Data Availability

Not applicable.
